# Writing for the reader: Using reader expectation principles to maximize clarity

**DOI:** 10.1007/s40037-022-00708-w

**Published:** 2022-03-08

**Authors:** Lorelei Lingard

**Affiliations:** Centre for Education Research & Innovation, and Department of Medicine, Schulich School of Medicine & Dentistry and Faculty of Education, Western University, London, Canada

In the Writer’s Craft section we offer simple tips to improve your writing in one of three areas: Energy, Clarity and Persuasiveness. Each entry focuses on a key writing feature or strategy, illustrates how it commonly goes wrong, teaches the grammatical underpinnings necessary to understand it and offers suggestions to wield it effectively. We encourage readers to share comments on or suggestions for this section on Twitter, using the hashtag: #how’syourwriting?

Clarity is a prerequisite for everything else a writer is trying to achieve. Aiming for conversational prose? Trying to write lyrically? Mounting a scathing argument? Crafting a subtle one? None are within reach unless the prose is first clear. But if clarity is the cornerstone of everything else we’re trying to achieve, then what’s the cornerstone of clarity?

The standard admonitions about clarity in academic writing include: “Write shorter sentences!”, “Avoid passive voice!”, and “Limit unnecessary jargon!” The trouble is that such advice is insufficient: you can follow these rules and still lose your reader in boggy prose. That’s because the cornerstone of clarity is not just word number, or word choice, or word meaning—it is also word *location*.

This Writer’s Craft distils the reader expectation approach for achieving clarity through attention to the structural location of information [1–3]. Drawing on work from the fields of rhetoric, linguistics and cognitive psychology, the reader expectation approach reflects this premise: readers of English interpret information more easily if it is placed where they expect to find it. Reader expectations about structure, therefore, constitute a powerful resource for writers. This Writer’s Craft outlines the key principles of reader expectation and illustrates how to apply them in sentences and paragraphs to achieve those coveted prose features: clarity and flow.

## The reader expectation approach

The central concept of reader expectation is that readers of English know “*where* to look for *what*” [2]. Location governs meaning and interpretation, because “*where *a word appears in a sentence will control most of the *functions *to which it will be put” [2, p. 36]. As readers of English, many of us know these expectations intuitively—it is how we make sense when we read.^1^ But as writers, we need to make them explicit and conscious. Four principles for harnessing reader expectation can help with that project.

### State the topic early

Readers expect that whatever shows up first in a sentence is the main idea. It establishes their perspective for viewing the sentence as a coherent unit. Therefore, writers should imagine the reader beginning each sentence asking “whose story is this?” By giving them that information early, they allow the reader to open a cognitive folder to organize all the details that will follow. This is most effectively achieved by placing the main character (person or thing) as the subject of the main clause. Because the* subject position* is one of the strongest meaning slots in a sentence [4], using it in this way powerfully controls the reader’s perspective.

*Supervisors* are reluctant to fail struggling trainees.

This sentence is about supervisors. If it were about struggling trainees, we’d need to flip it so that the trainees come first:

*Struggling trainees* are rarely failed by supervisors.

This is why the admonition to “Avoid passive voice!” is problematic: here we need the passive voice to satisfy the reader’s expectation that the first character to appear is the main one.

It’s not enough just to put the main character in the subject position, however; you also need to ensure that the subject position comes early in the sentence. The following example opens with a wind-up [5], a ***prepositional phrase string*** before the main subject:

***According to years of research in workplace-based training environments and largely unchanged by numerous faculty development efforts***, *supervisors* are reluctant to fail struggling trainees.

The wind-up has the effect of delaying the subject which keeps the reader searching for the answer to “whose story is this?” until more than halfway through the sentence. When readers’ expectations are deferred in this way, they may either get lost in the prepositional phrase because they lack a topic folder to house these details, or they may settle on one of the words in the prepositional phrase as the main character (like “workplace-based training” or “faculty development”). Either way, you have the ingredients for confusion rather than clarity. An easy solution is to put the detail after the main subject:

*Supervisory reluctance* to fail struggling trainees is documented by years of research in clinical training environments, and remains largely unchanged by faculty development efforts.

### Follow the main subject closely by its verb

Once readers know the topic, they wonder “what’s happening?” to that person or thing. The verb provides this action, so readers “lean forward” [2, p. 44] to it. Seeking what’s called “syntactic resolution” [3, p. 4], readers of English expect the verb to follow the subject, if not immediately then very soon. Because of this expectation, material “that intervenes between subject and verb is read as an interruption” [3, p. 4]. Readers resist recognizing interrupting material as important because its structural location, in the liminal space between subject and verb, brands it as incidental. Knowing this reader expectation, writers should keep *subject* and ***verb*** close together, only interrupting if it achieves a particular purpose, and not more than a short phrase. The longer *the interrupting material*, the more likely it will contain something important that might get glossed over as the reader leans to the verb, as in this example:

*Clinical supervisors*, because of their fears for patient safety and their frustration with trainees’ repeated mistakes during clinical performances, ***may not respond*** in a fair and measured manner to struggling trainees.

The following revision moves the ***verb*** beside its *subject*:

*Clinical supervisors* ***may not respond*** in a fair and measured manner to struggling trainees, because of their fears for patient safety and frustration about trainees’ repeated mistakes during clinical performances.

This version provides immediate syntactic resolution, freeing the reader up to focus on other interpretative details as the sentence unfolds.

### Use the stress position to emphasize new information

The main subject position is not the only powerful meaning slot in a sentence. Readers also expect extra emphasis whenever they come to a stop. This structural location is called a “stress position” [2, p. 59]. Any moment of full syntactic closure creates a stress position [3], meaning that the reader recognizes they are reading the last part of the grammatical structure, whether clause or sentence. Primary stress positions occur at the end of the sentence; secondary stress positions can also be created mid-sentence through the proper use of colons or semi-colons.

Stress positions offer the writer a golden opportunity to introduce a new idea of importance. But writers often get this wrong, putting incidental information in stress positions and placing important information elsewhere. In fact, Gopen judges that the misplacement of stress-worthy information is the primary enemy of clear writing [2, p. 58]. To judge whether such misplacement plagues your own writing, highlight the words you intend to stress with red font and then check—are they occupying a stress position or not? If not, you’re frustrating readers’ expectations, and they may lose the thread.

Consider this example:

Clinical supervisors reported experiencing frustration, fear for patient safety, and even anger when their trainees repeatedly struggled.

The stress position is taken up by the idea of “trainees repeatedly struggled”, which is fine if that’s what the writer intended. If, however, they wanted to emphasize “anger” (which is suggested by the construction “even anger”), they have misplaced that information. It follows a comma as part of a list; it does not occupy the stress position at the end of the sentence. Commas do not create stress positions, so the reader won’t readily discern that “anger” is the climax (the important new information) in this sentence. The writer has tried to signal the emphasis by adding “***even*** anger”, but this signalling may not be sufficiently strong to ensure the reader doesn’t misinterpret. The following revisions use the stress position to signal that “anger” is the important new information in this sentence:

Clinical supervisors reported that, in addition to frustration and fear for patient safety with struggling trainees, they also experienced anger.

Clinical supervisors reported that, in addition to frustration and fear for patient safety with struggling trainees, they also experienced another emotion: anger.

Note, that stress can be amped up: the second revision uses both a colon and a period to intensify the emphasis. No reader could miss this climax!

Compound and complex sentences may contain both primary and secondary stress positions, making them useful when more than one piece of important new information is being introduced. Consider the following example which offers four ***stress positions***:

Fatigue colours ***all relationships during clinical training***: it shapes residents’ ***interactions with patients***; it infects their ***families and friendships***; it can even distort their sense of ***identity and self-worth***.

The first three are secondary stress positions, created by a colon and semi-colons. The last stress position in the sentence is the primary one, just before the final period. Therefore, although there are four important pieces of new information in the sentence, what we should leave with is the expectation that the last—“sense of identity and self-worth”—is primary. Usually this means that this material is what will be developed next.

### Signal backward and forward links between sentences

This idea of development from one sentence to the next brings us to the final principle. Readers do not read sentences in isolation from one another. When a sentence ends, they leave with a sense of what to carry forward. When a new sentence begins, they expect a backward connection to what came immediately before [2, p. 49]. Such backward and forward links are the key to achieving flow: a coherent thread of logic that weaves across sentences and paragraphs.

Consider the sentence: “Equity and inclusion in postgraduate training cannot be reduced to markers such as representation during selection processes.” This sentence could lead in a number of directions, so readers leaving it and entering the next will be asking themselves which of “equity”, “inclusion”, “postgraduate training”, “markers” and “selection processes” will appear in the new sentence to form a logical connection. Imagine that the next sentence is: “The difficult terrain of assessment must also be considered.” There is only that little word “also” to signal connection—but with which of the preceding ideas? Now imagine instead that the next sentence is: “Such reduction constrains the real and lasting change we need to ensure a robust and diverse health workforce.” Here, the phrase “such reduction” backward signals to tell us what idea from before we’re developing in the new sentence.

As this example illustrates, the sense of backward linkage comes from reusing material that the reader has already seen. This follows the linguistic principle of “Given-New”, which says that we should express known information (the “given”) before previously unknown information (the “new”) [6]. Consistently putting “given” information from the preceding sentence into the main subject position of the next sentences creates for the reader a continuous flow of thought. Consider this published example, with *given* and ***new*** highlighted:

***Peer review*** can sting. *It* is intended as a collegial, respectful enterprise, but ***the popular “Reviewer 2” meme in social media suggests that it often feels otherwise.*** *Reviewer 2 *symbolizes the ***peer reviewer who is rude, vague, smug, committed to pet issues, theories, and methodologies, and unwilling to treat the authors as peers.**** A recent linguistic analysis of such reviews* found features such as attitude markers (e.g., verbs like “reject”, sentence adverbs like “absurdly”, and adjectives like “illogical”), self-mention (e.g., “I cannot possibly imagine”), and boosters (e.g., “the manuscript is utterly ridiculous”) [7, p. 299]

Peer review is new in the first sentence. It becomes given, converted to the pronoun “it”, in the main subject position of the next sentence, which uses the final stress position to introduce something new: the Reviewer 2 concept. That idea then becomes given in the subject position of the third sentence, which ends with new information about the characteristics of peer reviews. Notice that the fourth sentence begins with a new idea, “A recent linguistic analysis”, but anchors it with a reference to the given “such reviews” from the preceding sentence. Such chain-linking based on Given-New helps to create a sense of logical flow. If you receive the feedback “I don’t follow your logic in this passage”, check whether you are observing the Given-New principle and chain-linking your sentences to satisfy this reader expectation.

## Conclusion

Knowing the four main reader expectations can help writers harness the power of structural location in their manuscripts. Readers expect the main topic to provide context for the rest of the sentence, so provide it early, preferably in the main subject position. Readers lean forward to the verb, so avoid interrupting the subject-verb connection with other material. Readers expect important new information to appear at stress positions, so place such information carefully and don’t waste stress positions on incidental material. And, finally, readers leave and enter sentences trying to form connections between them, so make this easy for them with backward and forward linkages that observe the Given-New principle.

Reader expectation can be harnessed to improve clarity and logical flow in your writing. But don’t approach these strategies as rigid rules or assume that using them guards entirely against misinterpretation. Gopen and Swan warn that “*we cannot succeed in making even a single sentence mean one and only one thing; we can only increase the odds that a large majority of readers will tend to interpret our discourse according to our intentions*” [3, p. 7]. Readers have both agency and their own agendas. We cannot control them. But we can use their expectations. And sometimes we can do so in unexpected ways to good rhetorical effect. We may delay the main topic of the sentence for suspense; we may separate the subject and verb to add nuance; we may violate the Given-New principle to upset common assumptions. Writing rules are made to be broken, after all. Just not by accident.

## Footnotes

1 Gopen’s model is based on English speakers reading English prose. If we’re reading English as an additional language, which is the case for many readers in our scholarly field, these expectations may not be so intuitive. This makes reader expectation theory even more useful, as it can help non-native English writers anticipate English readers’ needs that may not feel intuitive to them.
